# Precursor exhausted CD8^+^T cells in colorectal cancer tissues associated with patient’s survival and immunotherapy responsiveness

**DOI:** 10.3389/fimmu.2024.1362140

**Published:** 2024-03-06

**Authors:** Hao Huang, Junwei Ge, Zhang Fang, Shaoxian Wu, Hongwei Jiang, Yanyan Lang, Junjun Chen, Wenlu Xiao, Bin Xu, Yingting Liu, Lujun Chen, Xiao Zheng, Jingting Jiang

**Affiliations:** ^1^ Department of Tumor Biological Treatment, The Third Affiliated Hospital of Soochow University, Jiangsu, Changzhou, China; ^2^ Jiangsu Engineering Research Center for Tumor Immunotherapy, The Third Affiliated Hospital of Soochow University, Jiangsu, Changzhou, China; ^3^ Institute of Cell Therapy, The Third Affiliated Hospital of Soochow University, Jiangsu, Changzhou, China

**Keywords:** precursor exhausted CD8^+^T cells, colorectal cancer, multi-color immunohistochemistry, multi-omics, immunotherapy

## Abstract

Exhausted CD8^+^T cells represent a distinct cellular lineage that emerges during both chronic infections and cancers. Recent studies have shown that persistent antigen exposure can drive the differentiation of precursor exhausted CD8^+^T cells, termed T_pex_ cells, which are characterized as TCF-1^+^PD-1^+^CD8^+^T cells. Elevated T_pex_ cell frequencies in the tumor microenvironment (TME) are associated with improved overall survival (OS) in cancer patients and heightened responsiveness to anti-PD-1 therapy. In our present study, we utilized multi-color immunohistochemistry (mIHC) to determine the localization and clinical implications of tumor-infiltrating T_pex_ cells within the TME of human colorectal cancer (CRC) tissues. We also conducted a multi-omics integrative analysis using single-cell RNA sequencing (scRNA-seq) data derived from both the murine MC38 tumor model and human CRC tissues. This analysis helped delineate the transcriptional and functional attributes of T_pex_ cells within the CRC TME. Furthermore, we employed spatial transcriptome sequencing data from CRC patients to investigate the interactions between T_pex_ cells and other immune cell subsets within the TME. In conclusion, our study not only established a method for T_pex_ cell detection using mIHC technology but also confirmed that assessing T_pex_ cells within the CRC TME could be indicative of patients’ survival. We further uncovered the transcriptional and functional characteristics of T_pex_ cells in the TME and ascertained their pivotal role in the efficacy of immunotherapy against CRC.

## Introduction

Immune checkpoint blockade (ICB) therapy, especially using PD-1 monoclonal antibodies (anti-PD-1), has emerged as a cornerstone in cancer immunotherapy because of its remarkable clinical efficacy (1, 2). Yet, challenges persist. Tumor heterogeneity and immune suppression within the tumor microenvironment (TME) impede the effectiveness of anti-PD-1 therapy, limiting its clinical response. Addressing these challenges to enhance ICB therapy can herald a new era in cancer immunotherapy.

The efficacy of anti-PD-1 therapy is anchored in its ability to restore CD8^+^T cell function and counteract immunosuppression within the TME (3, 4). However, many patients still do not respond to anti-PD-1 therapy. This unresponsiveness primarily stems from the inability to activate dysfunctional CD8^+^T cells within the TME, commonly known as primary exhausted T cells (T_ex_) (5, 6). CD8^+^T cells are instrumental in eradicating tumors and impeding their progression, acting as the primary effector cells in the host’s immune response against cancer. Their exceptional ability allows them to recognize and directly eliminate target cells by detecting antigen signals presented via MHC class I molecules (7). Our earlier work has highlighted that the infiltration density and functional status of CD8^+^T cells within the TME can serve as critical indicators for patient survival and cancer progression (8–12).

Upon antigen stimulation and co-stimulatory signals, CD8^+^T cells typically undergo rapid proliferation, and subsequently differentiate into effector and memory CD8^+^T cells during acute infections (6, 13). Yet, within the milieu of chronic infections or the TME, CD8^+^T cells face continuous stimulation from inflammation or tumor antigens. This sustained engagement hinders their effector and memory functionalities. Over time, constant stimulation propels CD8^+^T cells first into the precursor exhausted (T_pex_) stage and ultimately into the terminally differentiated exhausted (T_ex_) stage (6). T_pex_ cells are primarily characterized as TCF1^+^PD-1^+^CD8^+^T cells. Notably, these T_pex_ cells often exhibit elevated levels of activation markers like CD69 and CD107, inhibitory immune checkpoint molecules including LAG3 and CD200, and transcription factors such as ID3, EOMES, and T-bet (13).

Given the intermediate PD-1 expression levels in T_pex_ cells, recent studies have increasingly focused on examining their presence within tumor tissues. This interest is primarily due to their intrinsic connection to the therapeutic efficacy of anti-PD-1 treatments in cancer contexts (13–15). For instance, a study employing single-cell RNA sequencing (scRNA-seq) on tissues from 48 patients with metastatic melanoma, which included 17 responders and 31 non-responders to anti-PD-1 treatment, has revealed that tumor-infiltrating CD8^+^T cells can be broadly classified into early activation, memory, effector, and exhausted states. Notably, a higher count of TCF-1^high^CD8^+^ tumor-infiltrating lymphocytes (TILs) in tumor tissues is indicative of a more favorable response to anti-PD-1 therapy (14).

In our current study, our aimed to determine the localization and clinical significance of tumor-infiltrating T_pex_ cells within the TME of human colorectal cancer (CRC) tissues. Utilizing scRNA-seq data from both the murine MC38 tumor model and human CRC tissues, we elucidated the transcriptional and functional attributes of T_pex_ cells within this specific TME. Additionally, we harnessed spatial transcriptome (ST) sequencing data from CRC patients to explore how T_pex_ cells interacted with other cellular entities in the TME. Overall, our study not only pioneered a method for detecting T_pex_ cells using multi-color immunohistochemistry (mIHC) but also underscored the value of evaluating T_pex_ cells within the CRC TME as a potent prognostic indicator. Delving into multiple scRNA-seq datasets, we further discerned the unique features of T_pex_ cells and highlighted their pivotal role in determining the efficacy of immunotherapy in CRC patients.

## Materials and methods

### Patients and tissue specimens

The human CRC tissue microarray (TMA, catalog: HColA180Su16) was sourced from Shanghai Outdo Biotech Co., Ltd. (Shanghai, China). Specifically, the CRC TMA consisted of samples from 104 patients, namely 58 males and 46 females, ranging in age from 24 to 90 years. One patient was excluded due to the absence of clinical data. Survival data for all 104 patients were compiled and employed in the survival analysis. This study, which involved human participants, was reviewed and received approval from the Clinical Research Ethics Committee at Outdo Biotech (Shanghai, China, SHYJS-CP-1707004).

### mIHC staining

The mIHC staining was conducted using the Opal 7-color Manual IHC kit (Catalog No. NEL801001KT, PerkinElmer, USA), in conjunction with automated quantitative analysis tools from PerkinElmer, USA, according to the manufacturer’s instructions, consistent with our previously reported methodology (12). The primary objective of our current mIHC staining study was to identify cytokeratin (CK), CD8^+^T cells (CD8), activated T cells (PD-1), naïve/memory cells (TCF1), and proliferating cells (KI67) within CRC tissues and their adjacent normal counterparts. Nuclei were stained using 4’,6-diamidino-2-phenylindole (DAPI). The CRC TMA was first dried at 63°C for 1 hour. Subsequently, it was dewaxed in xylene and then rehydrated through a series of graded ethanol solutions. For antigen retrieval, we used a 1 mM EDTA solution at pH 9.0. After this, the tissue sections were allowed to cool at room temperature for 1 hour. Each section was treated with 100-200 µL of blocking solution and left to sit at room temperature for 15 minutes. We employed the following primary antibodies: anti-CD8 (catalog No. PA067, BioDot, USA), anti-TCF1 (1:50 dilution, catalog No. 2203, CST, USA), anti-PD-1 (catalog No. PA153, BioDot, USA), anti-KI67 (catalog No. GM724007, Gene Tech, China), and anti-CK (1:10 dilution, catalog No. PA125, BioDot, USA). The CRC TMA slide was then incubated with HRP-conjugated secondary antibodies (PerkinElmer, USA) in an Opal working solution (PerkinElmer, USA). The slide was mounted with ProLong Diamond Antifade Reagent with DAPI (Thermofisher, USA).

### Imaging analysis

Firstly, panoramic multispectral scanning of the slides was conducted using the TissueFAXS system (Tissue Gnostics Asia Pacific Limited, Austria). The images obtained were then processed with the StrataQuest analysis software (Version No.7.0.1.165, Tissue Gnostics Asia Pacific Limited, Austria). In this software, each fluorophore was spectrally separated into individual channels and archived as distinct files. The DAPI staining served as a guide, ensuring precise differentiation between each cell’s nucleus, cytoplasm, and cell membrane. The expressions of CK, CD8, TCF1, PD-1, and KI67 were integrated with DAPI staining, resulting in binary masks highlighting cells that exhibited these specified biomarkers. Ultimately, the CK binary mask was analyzed to enumerate local tumor cells. For tissue recognition, Pan-CK was employed to segregate stromal nuclei from epithelial tumor tissue regions.

### Bulk RNA-seq data analysis

For the TCGA-COAD cohort, the RNA-seq and clinical data were downloaded using the TCGAbiolinks R package. The consensus molecular subtypes (CMS) were predicted from the count matrix using the CMScaller package. Only patients with assigned CMS groups underwent further analysis. The Gene Set Variation Analysis (GSVA) for the CD8^+^T cell subsets was executed using the GSVA package, with the enrichment analysis results visualized through ggplot2 package. The GSVA also performed enrichment analysis for selected chemokines (*CXCL9/10/11*) and their receptor (*CXCR3*). To determine the cutoff point for feature genes from the enrichment analysis, the *surv_cutpoint* function from the survminer package was applied. Based on this cutoff, patients were categorized into two groups: “High score” and “Low score”. Kaplan-Meier survival analysis was subsequently conducted using the survival package, and results were visualized with the *ggsurvplot* function.

Regarding data from the Synapse platform (Synapse: syn2623706) (16), gene IDs were mapped to symbols via the idmap2 R package. To mitigate potential batch effects among datasets, the *ComBat* function from the sva package was deployed. CMS predictions for each sample were executed using the CMSclassifier package. Patients without CMS assignments were excluded. The ensuing analyses mirrored those performed for the TCGA-COAD cohort. For rectal cancer patients with incomplete or complete responses to neo-adjuvant therapy (17), the GSVA was utilized to score CD8^+^T cell subsets, and the results were visualized with ggplot2.

### scRNA-seq data analysis

Whole tumor data files for human CRC scRNA-seq were sourced from the Figshare website (https://figshare.com/articles/dataset/CRC_CD45_rda/14820318). We employed an unsupervised graph-based clustering algorithm through Seurat v4 (18) to categorize each cell by its gene expression profile. Diverging from the official *FindVariableFeatures* function, the R package DUBStepR (with default parameters) was employed to pinpoint highly variable genes. Cell cycling scores were determined via the *CellCycleScore* function, leveraging the gene set of the S and G2M phases from Seurat. The *ScaleData* function was then implemented to offset cell cycle effects in ensuing analyses. Principal component analysis (PCA) on the distinguished highly variable genes was undertaken, with the Harmony package being employed to mitigate batch effects. Dimensionality was reduced using the *RunUMAP* function on 30 principal components at a resolution of 0.1. Cells were then annotated in alignment with the clustering outcomes outlined in the article. Similarly, CD45^+^ data files for mouse MC38 scRNA-seq were retrieved from the Figshare website (https://figshare.com/articles/dataset/CRC_CD45_rda/14820318). The clustering methodology was analogous to that applied to the human scRNA-seq dataset. Data files pertaining to CD8^+^T cells for human CRC scRNA-seq were accessed from GEO DataSets (GSE108989). Following official protocols, we conducted unsupervised clustering. Cell annotation was based on the clustering results described in the article. The entire tumor data set for human CRC scRNA-seq, classified by CMS, was procured from the Synapse platform (Synapse: syn26844071) upon request. Clustering adhered to standard protocols, and cell annotation was reliant on the R package scibetR, using pre-processed scRNA-seq data as a reference.

Scores for CD8^+^T cells in treatment-related scRNA-seq datasets, specifically from colorectal and melanoma tumors (GSE205506 and GSE120575), were deduced via the *AddModuleScore* function. Subsequent visualizations were achieved using the R package ggplot2.

### Transcriptional regulatory network analysis

We conducted a transcriptional regulatory network analysis on CD8^+^T cells extracted from the mouse MC38 scRNA-seq dataset. Initially, the co-expression network was computed using GRNBoost2, available in the Python package pySCENIC. Subsequently, regulons were discerned utilizing the R package RcisTarget. The activity of each regulon was quantified for every cell through the R package AUCell. For dimensionality reduction of regulon activity, we adopted t-distributed stochastic neighbor embedding (t-SNE), executed via the *runSCENIC_3_scoreCells* function in the SCENIC R package (19). Visualization of the area under the curve (AUC) values for the regulons was facilitated using the *DoHeatmap* function within Seurat.

### Cell-cell interactions analysis

The scRNA-seq data from human CRC was classified by CMS and annotated using the R package, scibetR. These data were then employed to infer cell-cell interactions. Notably, the TAM-C1QC and CD8-GZMK cells were singled out for further analysis. To identify potential ligand/receptor interactions, we utilized the *statistical_analysis* function from the cellphonedb method, keeping the default parameters. The interactions identified were subsequently visualized using the *dot_plot* function from cellphonedb.

### Spatial transcriptome sequencing data analysis

Spatial transcriptome sequencing data were downloaded according to the article (20). The data files were processed following the tutorial created by Åsa Björklund & Paulo Czarnewski (https://nbisweden.github.io/workshop-scRNAseq/labs/compiled/seurat/seurat_07_spatial.html

#Deconvolution). Then, we deconvoluted the spatially-indexed datasets by R package SCDC (21) and predicted single-cell distribution according to previously processed single-cell data.

### Survival and multivariate Cox’s model analysis

According to the median value, the proportions of each subset in CRC TMA were divided into two groups: “Expression low” and “Expression high”. Subsequently, Kaplan-Meier survival curves and univariate Cox’s regression was calculated. Additionally, the correlation between CK subsets and CD8^+^T cell subsets was performed using GraphPad Prism 9 software. To further investigate the relationship among these variables and clinical data, multivariate Cox’s regression analysis was conducted using the *coxph* function from the R package survival. The result was visualized by R package forestplot.

### Statistical analysis

Statistical analyses for Kaplan-Meier survival curves and univariate Cox’s regression were conducted using GraphPad Prism 9 software. Multivariate Cox’s regression, bulk RNA-seq, scRNA-seq, and ST data analyses were performed with R version 4.2.2 and RStudio software. The pySCENIC tool, a lightning-fast Python implementation of GRNBoost2, was executed using Python 3.7.6. Specific details about the significance tests can be found in the accompanying figure legends.

## Results

### T_pex_ cell can be found in TME in both human and mouse CRC tissues

T_pex_ cells represent a unique subset of CD8^+^T cells, displaying traits reminiscent of both naïve and effector/exhausted cells. At the transcriptional level, T_pex_ cells preserve certain “stemness” attributes seen in naïve cells while concurrently showcasing partial effector functionalities typical of effector and exhausted cells (22). To delve deeper into the role of T_pex_ cells within the TME of CRC, we utilized single-cell transcriptome data from publicly accessible databases, covering both human and mouse samples (23, 24). This enabled us to scrutinize the gene expression and transcriptional regulation profile of T_pex_ cells. By leveraging on these scRNA-seq datasets, our aim was to identify the molecular signatures and controlling mechanisms governing T_pex_ cells in the CRC TME.

**Figure 1:**
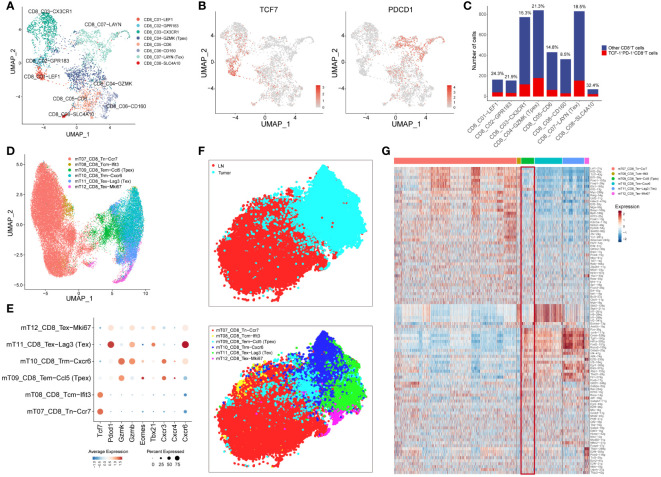
T_pex_ cell can be found in TME in both human and mouse CRC tissues **(A)** UMAP displayed CD8^+^T cell subsets in CRC tissue based on the clustering results described in the article. **(B)** UMAP showed the expression of *TCF7* and *PDCD1* in CD8^+^T cell subsets. **(C)** The bar plot displayed the number of TCF-1^+^PD-1^+^CD8^+^T cells in CD8^+^T cell subsets, and the percentages above the bars represent the percentages of TCF-1^+^PD-1^+^CD8^+^T cells among CD8^+^T cell subsets. **(D)** UMAP displayed CD8^+^T cell subsets in the mouse MC38 model based on the clustering results described in the article. **(E)** The dot plot displayed the expression of selected genes in CD8^+^T cell subsets. **(F)** t-SNE showed the location and clusters of CD8^+^T cell subsets, conducted by AUC values of regulons. **(G)** Heatmap showed AUC values of regulons in all CD8^+^T cell subsets.

Initiating our investigation with human CRC scRNA-seq data, we observed from clustering results highlighted in the referenced article that CD8^+^T cells could be segmented into eight distinct subgroups (**Figure 1A**). Significantly, hallmark genes of T_pex_ cell (22), such as *TCF7* and *PDCD1*, were chiefly co-expressed within the CD8_C04-GZMK subgroup (**Figures 1B, C**). This observation reaffirmed that the CD8_C04-GZMK cluster epitomized the T_pex_ cell subset. This finding aligned well with earlier studies indicating that T_pex_ cells predominantly express the granule-associated gene *GZMK* over *GZMB* (25).

Furthermore, we expanded our inquiry using scRNA-seq data from the mouse MC38 model. Relying on the clustering results outlined in the article, CD8^+^T cells were categorized into six distinct subgroups (**Figure 1D**). Importantly, the genes *Tcf7*, *Pdcd1*, and *Gzmk* were predominantly co-expressed within the mT09_CD8_Tem-Ccl5 subgroup, suggesting that this group exemplified the T_pex_ cell subset. In addition, the transcription factor *Eomes* was highly expressed in the T_pex_ cell subset, whereas *Tbx21* had increased expression in the T_ex_ subset (25). Analyzing chemokine receptor expression revealed that the receptor *Cxcr3*, linked to T-cell recruitment, was up-regulated in the T_pex_ cell subset. Simultaneously, the receptor *Cxcr4*, associated with circulation, experienced a similar up-regulation. In contrast, the receptor *Cxcr6*, tied to T-cell residency (26), manifested a reduced expression within the T_pex_ cell subset (**Figure 1E**).

SCENIC analysis showed marked discrepancies in transcriptional regulation between lymph node (LN) and TILs. Moreover, specific T-cell populations presented diverse transcriptional landscapes, with the T_pex_ cell subset (mT09_CD8_Tem-Ccl5) exhibiting a transitional state between LN and TILs (**Figure 1F**). The heatmap detailing AUC values of regulons indicated that T_pex_ cells bore resemblances to naïve CD8^+^T cells concerning naive attributes, while it mirrored the T_ex_ cell subset regarding exhaustion characteristics (**Figure 1G**). This finding underscored that T_pex_ cells were discernible in both human and mouse CRC TMEs.

### The proportions of activated and proliferating CD8^+^T cells are positively associated with the prognosis of CRC patients

**Figure 2:**
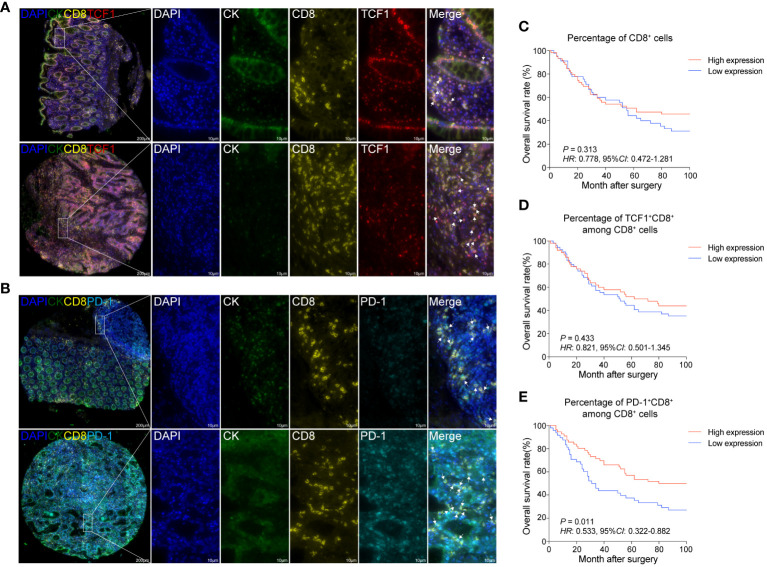
The proportions of activated and proliferating CD8^+^T cells are positively associated with the prognosis of CRC patients **(A)** mIHC and single-color images were obtained from CRC TMA, staining TCF1^+^CD8^+^T cells. DAPI: blue; CK: dark green; CD8: yellow; TCF1: red. **(B)** mIHC and single-color images were obtained from human CRC TMA, staining PD-1^+^CD8^+^T cells. DAPI: light blue; CK: dark green; PD-1: sky-blue. **(C)** Kaplan-Meier survival analysis of the percentage of CD8^+^T cells in CRC patients. **(D)** Kaplan-Meier survival analysis percentage of TCF1^+^CD8^+^T cell among CD8^+^T cells in CRC patients. **(E)** Kaplan-Meier survival analysis percentage of PD-1^+^CD8^+^T cell among CD8^+^T cells in CRC patients. **(C–E)** The high-expression group and low-expression group were defined by the median values of percentages and the *P* values for the differences between the high- and low-expression groups were calculated by using the Kaplan-Meier test, whereas *HR* and 95%*CI* using univariate Cox’s regression.

To bolster our findings, we performed mIHC staining on both CRC tissues and normal colorectal tissues. This enabled us to discern the spatial distribution and proportions of various CD8^+^T cell subsets: overall CD8^+^T cells, TCF1^+^CD8^+^T cells (indicative of naïve/memory cells), PD-1^+^CD8^+^T cells (representing activated cells), and KI67^+^CD8^+^T cells (denoting proliferating cells) (**Figures 2A, B**; **Supplementary Figures 1**, **2A**, **3A**).

Contrasting the cancerous tissues with their normal counterparts, we observed a reduction in the proportion of CD8^+^T cells within the cancer tissues. Among the CD8^+^T cell subsets, the frequency of TCF1^+^CD8^+^T cells exhibited a decline, while that of KI67^+^CD8^+^T cells experienced an uptick. The proportion of PD-1^+^CD8^+^T cells, however, remained relatively unchanged (**Supplementary Figure 3A**).

Our survival analysis revealed that the presence ratios of CD8^+^T cells and TCF1^+^CD8^+^T cells in tumor tissues were not significantly associated with patient outcomes (**Figures 2C, D**). Intriguingly, a heightened presence of PD-1^+^CD8^+^T cells and KI67^+^CD8^+^T cells was positively correlated with improved patient prognosis (**Figure 2E**; **Supplementary Figure 2B**). Thus, it could be inferred that increased infiltration of activated and proliferating CD8^+^T cells augmented the prognosis for CRC patients.

### Increased T_pex_ cell infiltration is associated with the improved prognosis of CRC patients

**Figure 3:**
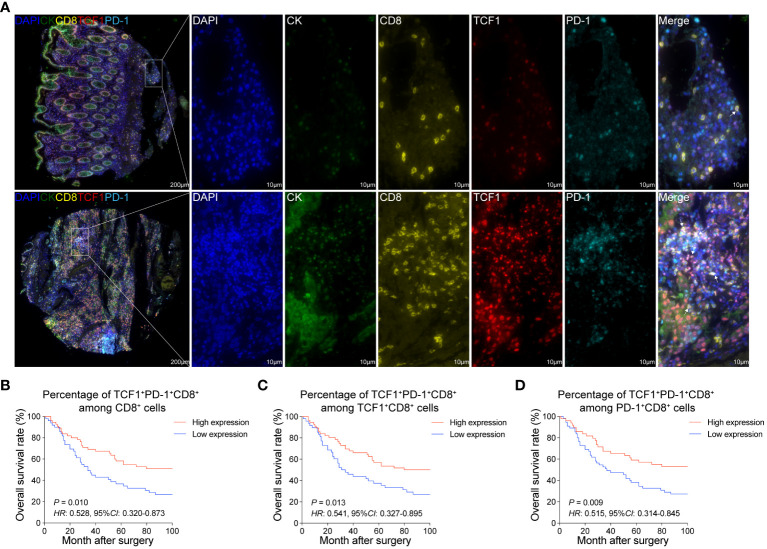
Increased T_pex_ cell infiltration is associated with the improved prognosis of CRC patients **(A)** mIHC and single-color images were obtained from CRC TMA, staining TCF1^+^PD-1^+^CD8^+^T cells. DAPI: blue, CK: dark green, CD8: yellow, TCF1: red, PD-1: sky blue. **(B)** Kaplan-Meier survival analysis of the percentage of TCF1^+^PD-1^+^CD8^+^T cell among CD8^+^T cells in CRC patients. **(C)** Kaplan-Meier survival analysis of the percentage of TCF1^+^PD-1^+^CD8^+^T cell among TCF1^+^CD8^+^T cells in CRC patients. **(D)** Kaplan-Meier survival analysis of the percentage of TCF1^+^PD-1^+^CD8^+^T cell among PD-1^+^CD8^+^T cells in CRC patients. **(B–D)** The high-expression group and low-expression group were defined by the median values of percentages, and the *P* values for the differences between the high- and low-expression groups were calculated by using the Kaplan-Meier test, whereas *HR* and 95%*CI* using univariate Cox’s regression.

Distinct from other CD8^+^T cell subsets, the spatial distribution of T_pex_ cells may be intrinsically tied to their biological role. To delve into this, we employed mIHC staining on CRC tissues and their adjacent normal tissues (**Supplementary Figure 1**). This allowed us to scrutinize the spatial distribution and ratios of TCF1^+^PD-1^+^CD8^+^T cells (referred to as T_pex_ cells) and KI67^+^TCF1^+^PD-1^+^CD8^+^T cells (proliferating T_pex_ cells) (**Figure 3A**; **Supplementary Figure 2C**).

Upon comparison, both T_pex_ cell and proliferating T_pex_ cell proportions in CRC tissues were broadly consistent with those in normal colorectal tissues. A notable exception was a marginal decrease in the proportion of proliferating T_pex_ cell within the overall T_pex_ cell population (**Supplementary Figures 3B, C**). Furthermore, survival analysis highlighted a positive correlation between the proportions of T_pex_ and proliferating T_pex_ cell, suggesting an association with improved patient outcomes (**Figures 3B–D**; **Supplementary Figures 2D–F**).

### Multivariate Cox’s model analysis of clinical characteristics and various infiltrates

**Figure 4:**
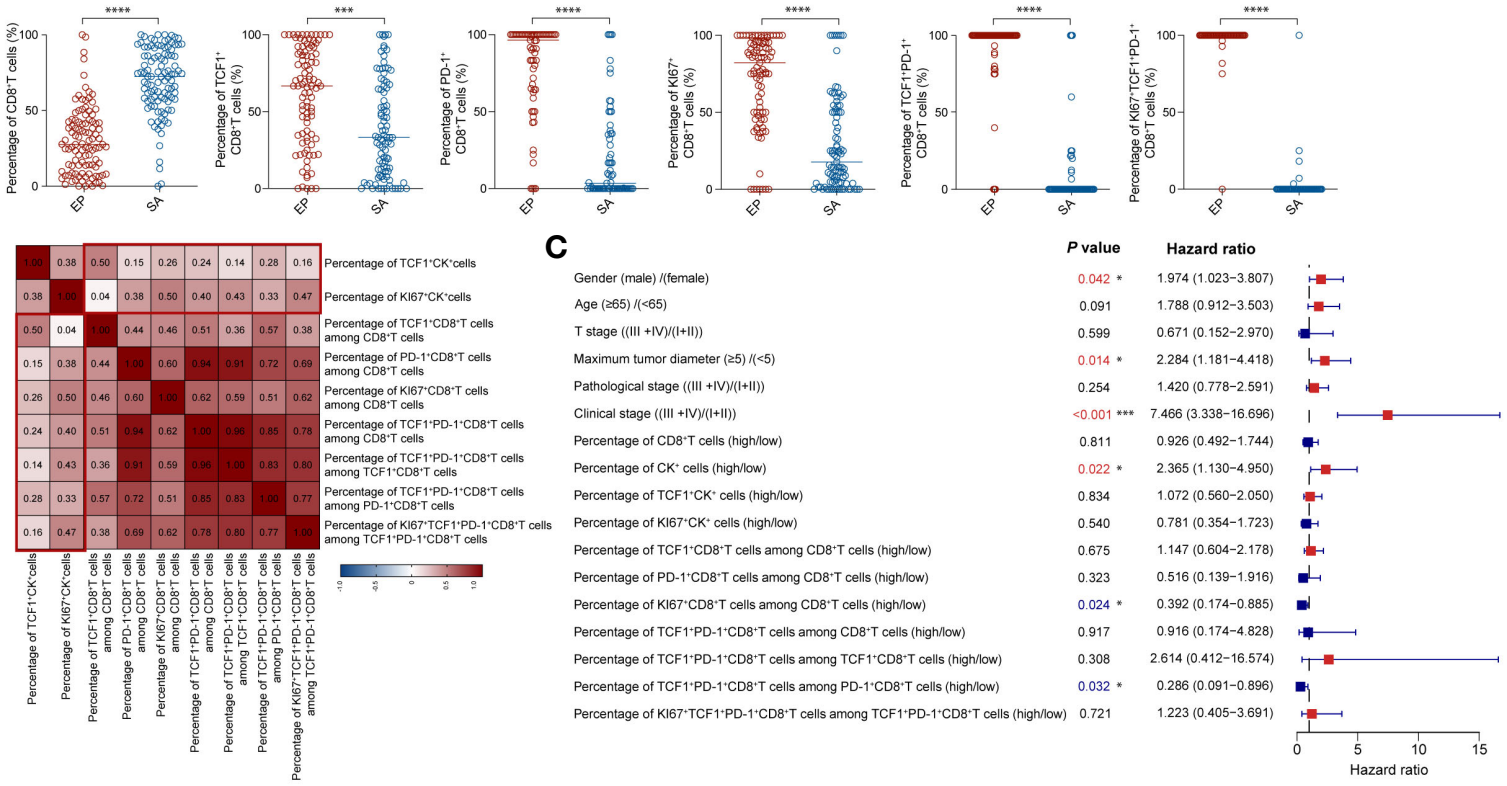
Multivariate Cox model analysis of clinical characteristics and different cellular infiltrations **(A)** Comparison of the proportions of CD8^+^T cell subsets between epithelial and stromal region in CRC tissues. **(B)** The correlation heatmap analyzed percentages of CK subsets and CD8^+^T cell subsets. **(C)** Forest plot showed results of multivariate Cox model analysis among clinical data and percentages of CK subsets and CD8^+^T cell subsets in CRC patients. **(A–C)** Statistical analyses in **(A)** were performed using a paired, two-tailed Student’s *t*-test. ^*^ *P* < 0.05, ^***^ *P* < 0.001, ^****^ *P* < 0.0001. The correlation coefficients in **(B)** were calculated by Spearman correlation. The *P* values, *HR*, and 95%*CI* in **(C)** were calculated by multivariate Cox’s regression.

To delve deeper into the distribution of CD8^+^T cell populations within tumor tissues, we compared the proportions of these populations in both epithelial and stromal regions. Our findings showed that the stromal region had a markedly higher overall proportion of CD8^+^T cells than the epithelial region. Yet, within the epithelial region itself, there was a significantly elevated proportion of activated, memory, and proliferating CD8^+^T cells when compared to their counterparts in the stromal region (**Figure 4A**).

As highlighted in earlier research (27), quiescent tumor cells often cluster together, and an increased proportion of these cells may inhibit immune cell infiltration. In contrast, a heightened presence of proliferating tumor cells can enhance immune cell infiltration. Notably, the balance between proliferating and quiescent tumor cells can be indicative of tumor immunogenicity.

To corroborate this, we delved into the spatial distribution and proportions of CK^+^, TCF1^+^CK^+^, and KI67^+^CK^+^ epithelial cells using mIHC staining (**Supplementary Figure 4A**). In alignment with prior findings, our analysis indicated that the ratios of CK^+^ and TCF1^+^CK^+^ epithelial cells didn’t considerably affect patient outcomes (**Supplementary Figures 4B, C**). However, patients with a greater proportion of KI67^+^CK^+^ epithelial cells presented with more favorable prognoses (**Supplementary Figure 4D**). Additionally, we explored the correlation between the proportions of TCF1^+^CK and KI67^+^CK epithelial cells and various CD8^+^T cell subsets. Interestingly, KI67^+^CK epithelial cells exhibited stronger correlations with most CD8^+^T cell subsets compared to TCF1^+^CK epithelial cells. The sole exception was the naïve/memory TCF1^+^CD8^+^T cells (**Figure 4B**).

Multivariate Cox’s regression analysis revealed several pivotal factors significantly impacting patient survival rates (**Figure 4C**). First, gender stood out as a critical determinant; male patients demonstrated a decreased survival rate. Additionally, the size of the tumor, specifically the maximum diameter, was inversely associated with survival: smaller tumors corresponded to better survival outcomes. Clinical stage was another influential factor, patients diagnosed at earlier tumor stages exhibited superior survival rates. Delving into cellular dynamics, varying levels of infiltrating immune cells emerge as significant. Specifically, a heightened proliferation of CD8^+^T cells and a greater proportion of activated CD8^+^T cells expressing TCF1 (percentage of T_pex_ cells within the PD-1^+^CD8^+^T cell cohort) positively correlated with improved survival, especially when juxtaposed against the proportion of TCF1^+^CK cells in the overall tumor cell population. In essence, our multivariate Cox’s regression analysis illuminated the key variables affecting patient survival rates, encompassing factors like gender, tumor dimensions, stage of tumor development, and cellular infiltration patterns. These insights bear significant value, offering clinicians a compass for tailoring treatment strategies and making well-informed patient management choices.

### The distribution of T_pex_ cells is positively correlated with TAM1 in TME

To explore the interactions between T_pex_ cells and other cell types within the TME, we turned to ST sequencing data from CRC patients (20). Analyzing proximity and co-localization patterns granted us a deeper understanding of potential cellular interactions and the specific micro-environmental niches T_pex_ cells occupies.

**Figure 5:**
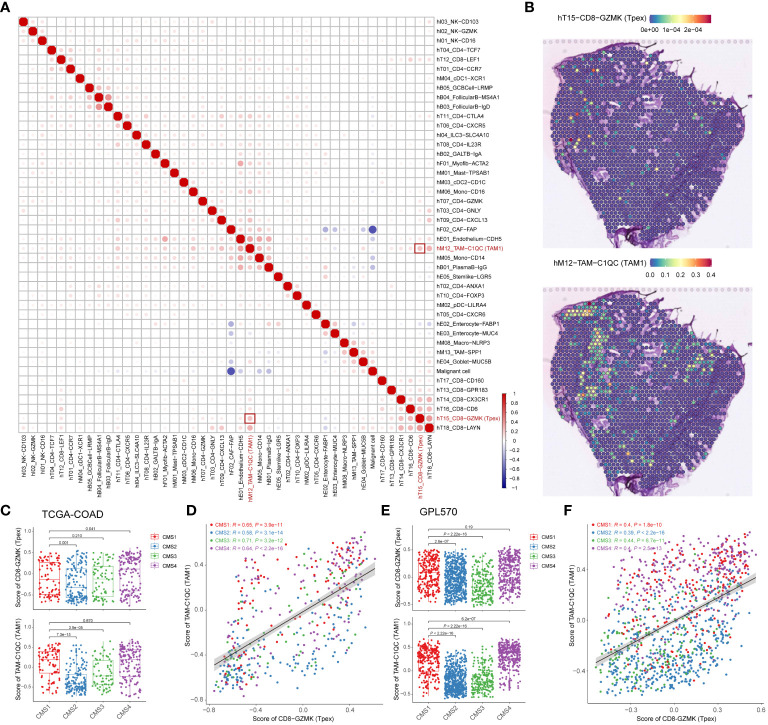
The distribution of T_pex_ cells is positively correlated with TAM1 in TME **(A)** The correlation heatmap analyzed percentages of deconvoluted clusters according to the referred scRNA-seq data. **(B)** Transcriptome maps showed deconvoluted clusters of TAM1 and T_pex_ cells. **(C)** The bar plot showed scores of TAM1 and T_pex_ cells in TCGA-COAD cohorts calculated by the gsva method, grouped by CMS classification. **(D)** The scatterplot showed the correlation between scores of TAM1 and T_pex_ cells in TCGA-COAD cohorts. **(E)** The bar plot showed scores of TAM1 and T_pex_ cells in CRC tissue data of the Synapse platform calculated by the gsva method, grouped by CMS classification. **(F)** The scatterplot showed the correlation between scores of TAM1 and T_pex_ cells in CRC tissue data of the Synapse platform. **(A–F)** The correlation coefficients in **(A)** were calculated by Spearman correlation. The correlation coefficients in **(D, F)** were calculated by Pearson correlation. Statistical analyses in **(C, E)** were performed using the Kruskal-Wallis multiple-comparison test.

To systematically assess correlations across various cell types, we deconvoluted spatially-indexed datasets (21) and predicted the distribution of individual cells (24). Notably, our findings unveiled a positive correlation between T_pex_ cells and TAM1 (specifically, the TAM-C1QC subset), hinting at a potential interplay between these cell groups (**Figure 5A**). To further elucidate this relationship, we projected the derived cell clusters onto a transcriptome map (**Figure 5B**; **Supplementary Figure 5**). This graphical representation enriched our grasp on the spatial interplay between T_pex_ cells and TAM1 within the TME.

CRC has been categorized into four distinct CMS based on gene expression profiles and their molecular characteristics (16). To delve deeper into the relationship between T_pex_ cells and TAM1, we analyzed their correlation using bulk RNA-seq data (16, 28) and computed immune infiltration scores. This analysis unveiled a marked correlation between T_pex_ cells and TAM1 (**Figures 5C–F**). Intriguingly, the association between T_pex_ cells and TAM1 appeared more robust in the CMS1 and CMS4 subtypes than in the CMS2 and CMS3 subtypes. This pointed towards a particularly potent interaction between T_pex_ cells and TAM1 in immunogenic tumors. In essence, our research highlighted the co-localization and potential interplay between T_pex_ cells and TAM1 within the TME. Furthermore, when viewed through the lens of CMS subtypes, it’s evident that the synergy between T_pex_ cells and TAM1 was especially pronounced in immunogenic tumors.

### T_pex_ cells is regulated by TAM1 through co-stimulatory and chemokine signaling

Through an integrated analysis of both spatial and bulk transcriptome sequencing data, we uncovered strong evidence of the interaction between T_pex_ cells and TAM1 within the TME. This emphasized their potential collaborative role in this context. Yet, the precise mechanisms driving their interaction remain to be elucidated. To delve deeper into this dynamic, we turned to scRNA-seq data from CRC, classified according to the CMS (29). Our aim in analyzing this dataset was to further understand the molecular intricacies and functional interplay between T_pex_ cells and TAM1. By undertaking this comprehensive analysis, we hope to illuminate the driving forces behind their interaction, enriching our understanding of the complex cellular dynamics at play within the TME.

**Figure 6:**
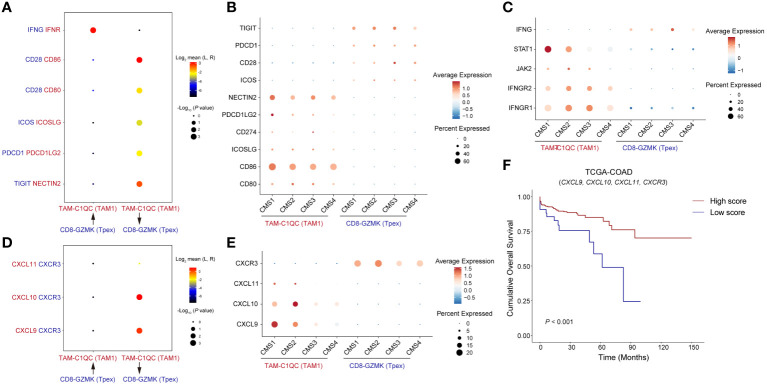
T_pex_ cells is regulated by TAM1 through co-stimulatory and chemokine signaling **(A)** The dot plot showed interactions of paired ligands and receptors. The dots’ color represents the mean values of paired ligands and receptors transformed by Log_2_. The dots’ size represents *P* values of interaction strength transformed by -Log_10_. **(B)** The dot plot showed the expression of ligands and receptors related to co-stimulatory signaling in **(A)**, grouped by CMS classification. **(C)** The dot plot showed the expression of ligands and receptors related to IFN-γ signaling in **(A)**, grouped by CMS classification. **(D)** The dot plot showed interactions of paired chemokines and chemokine receptors. **(E)** The dot plot showed the expression of chemokines and chemokine receptors in **(D)**, grouped by CMS classification. **(F)** Kaplan-Meier survival analysis score of genes showed in **(D)** among TCGA-COAD cohorts, the cutoff value was defined by *surv_cutpoint()* function in R package survminer.

Analysis of cell-cell communication revealed a pivotal role for the co-stimulatory molecules CD80/86-CD28 and ICOS-ICOSL in the interaction between T_pex_ cells and TAM1 (**Figure 6A**). Notably, the immune checkpoint molecules PD-1-PD-L2 and TIGIT-Nectin2 also exhibited significant enrichment. Within the different CMS groups, *CD86*, *PDCD1LG2* (PD-L2), and *NECTIN2* were over-expressed in the CMS1 group (**Figure 6B**). Moreover, IFN-γ, originating from T_pex_ cells, modulated TAM1 through its receptor (**Figure 6A**). The transcription factor *STAT1*, which is linked to the IFN-γ pathway, also showed heightened expression in the CMS1 group (**Figure 6C**). TAM1’s immune-regulatory function was not limited to merely producing inflammatory cytokines. It secreted chemokines like CXCL9, CXCL10, and CXCL11, all downstream effectors of the IFN-γ pathway, to attract T cells. Additionally, IFN-γ can induce the up-regulation of CXCR3 on CD8^+^T cells. Considering prior findings that highlight an increased expression of *CXCR3* on T_pex_ cells (**Figure 1D**), we postulated a potential interaction between TAM1 and T_pex_ cells via the CXCL9/10/11-CXCR3 axis. Our analyses also underscored a marked enrichment of the CXCL9/10-CXCR3 interaction between these cell populations (**Figure 6D**). Notably, the expression of *CXCL9* within the CMS1 group was significantly higher compared to other subtypes (**Figure 6E**). This aligned with the previously observed amplification of IFN-γ signaling. Importantly, heightened expression of these chemokines and receptors between TAM1 and T_pex_ cells was correlated with better prognoses in CRC patients (**Figure 6F**). In essence, the dynamic relationship between TAM1 and T_pex_ cells encompassed a blend of co-stimulatory molecules, immune checkpoint proteins, and downstream chemokines and their receptors, all integral to the IFN-γ signaling cascade.

### T_pex_ cells can predict the therapeutic potential of CRC immunotherapy

**Figure 7:**
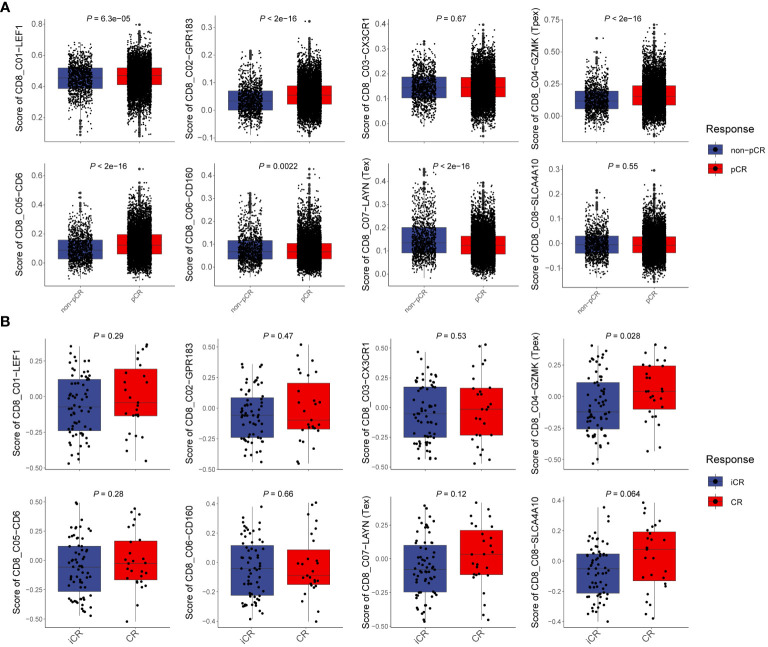
T_pex_ cells can predict the therapeutic potential of CRC immunotherapy **(A)** Boxplots showed the scores of CD8^+^T cell subsets in scRNA-seq data between non-complete response and complete response CRC patients, calculated by the gsva method. **(B)** Boxplots showed the scores of CD8^+^T cell subsets in bulk RNA-seq data between incomplete response and complete response rectal cancer patients, calculated by the gsva method. **(A, B)** Statistical analyses were performed using unpaired, two-tailed Student’s *t*-test.

CD8^+^T cells are cornerstone players in tumor immunotherapy. To delve deeper into the functionality of T_pex_ cells and its differentiation from other CD8^+^T cell subsets in the context of immunotherapy, we dissected characteristic gene sets derived from scRNA-seq data of CD8^+^T cells obtained from CRC patients. By integrating insights from both scRNA-seq (30, 31) and bulk RNA-seq (17) datasets related to immunotherapy, we computed scores for each CD8^+^T cell subset. Our initial focus was on analyzing CD8^+^T cell subsets from scRNA-seq datasets of CRC and melanoma patients. This involved evaluating specific scores for each subset (**Figure 7A**; **Supplementary Figure 6**). Notably, our statistical scrutiny unveiled significantly elevated T_pex_ cell scores in CRC and melanoma patients who showed positive responses to immunotherapy compared to those who did not. In stark contrast, the T_ex_ scores in responders from both patient groups were markedly reduced relative to non-responders. Furthermore, we navigated through bulk RNA-seq data sourced from rectal cancer patients undergoing neoadjuvant therapy (**Figure 7B**). Corroborating our earlier findings, the data highlighted that patients demonstrating a complete response to immunotherapy had noticeably heightened T_pex_ cell scores. In contrast, the T_ex_ cell score was markedly depressed in those showcasing an incomplete response. Collectively, these revelations underscored the potential of T_pex_ cell proportions as pivotal indicators for prognosticating the efficacy of immunotherapy targeting CD8^+^T cells in CRC.

## Discussion

T_ex_ cells undergo a complex differentiation process governed by an array of regulators, including key transcription factors like TOX, TCF-1, and T-bet, alongside crucial epigenetic modifiers such as METTL3, METTL14, and FTO (32–34). A hallmark of T_ex_ differentiation is the progressive attenuation of effector functionality. This manifests as reduced responsiveness to homeostatic cytokines like IL-7 and IL-15, persistent expression of inhibitory receptors, and significant alterations in both the transcriptomic and metabolomic landscapes. Such characteristics uniquely distinguish T_ex_ from T_em_ and T_mem_ cells, rendering them an essential player in immune dysfunction and diminished responsiveness to immunotherapy in oncological contexts (6, 32). Notably, a critical attribute of T_ex_ cells within the TME exhibit high levels of PD-1 and other inhibitory immune checkpoints, prompting the exploration of anti-PD-1 therapies and other ICB strategies to mitigate T cell exhaustion and rejuvenate the effector functions of CD8^+^T cells. The T_pex_ cells, a pivotal phase in CD8^+^T-cell differentiation, show particular sensitivity to anti-PD-1 therapy, highlighting their significance in therapeutic interventions (5, 13, 35).

Huang et al. have shed light on a distinct subset of CD8^+^T cells located in the tumor-draining lymph node (TdLN). These cells, characterized as TCF-1^+^TOX^-^ and termed tumor-specific memory CD8^+^T cells (T_TSM_), are endowed with pronounced anti-tumor capabilities (36). Furthermore, they stand out as a pivotal CD8^+^T-cell subset that responds aptly to PD-1/PD-L1 inhibitors (36). Given their inherent memory and sustained anti-tumor attributes, therapeutic interventions, such as adoptive T-cell therapy and anti-PD-1 therapy targeting T_TSM_ cells, may represent an emergent focal point in the realm of cancer immunotherapy research and clinical applications.

Delving into the molecular intricacies, the transcription factor TCF-1 emerges as a central regulator governing T_pex_ cells differentiation. Flow cytometry analyses have further substantiated this, distinguishing the TCF-1^+^Ly108^+^PD-1^+^ phenotype as representative of T_pex_ cells, which starkly contrasts with the KLRG1^+^CD39^+^Tim-3^+^ phenotype identified as T_eff_-like cells during the nascent phases of chronic infection and tumorigenesis (37). Another fascinating revelation is the influence exerted by overexpression of TCF-1, it augments the proportion of T_pex_ cells, thereby anchoring antigen-specific CD8^+^T cells in a prolonged stable state (37). TCF-1 further demonstrates its multifaceted roles by enhancing Bcl-2 expression via c-Myb, a mechanism that ultimately bolsters the survival of T_pex_ cells (38).

Gueguen et al. have employed an innovative approach to analyze TILs in primary non-small cell lung cancer (NSCLC) using scRNA-seq and TCR sequencing. This approach reveals nuanced insights into their functional organization (39). Two intriguing CD8^+^TIL subgroups come to the fore, each embodying distinct memory-like gene profiles: CD8-GZMK, indicative of circulating precursors found in the bloodstream, and CD8-XCL1, which signifies tissue-resident precursors within the cancerous tissue itself. A particularly noteworthy observation is the convergence of these precursors within tumor sites, where they evolve into what are commonly referred to as terminally differentiated or “exhausted” CD8-LAYN cells. The preferential TCR expansion observed in CD8-XCL1 over its CD8-KLF2/CD8-GZMK counterparts is promising, pointing towards improved outcomes in response to ICB (39).

Furthermore, accumulating evidence from multiple studies postulates that analysis of T_pex_ cells from TILs of NSCLC patients, both pre and post-treatment, can serve as a pivotal gauge for therapeutic efficacy (40, 41). Adding to this knowledge, our current investigation pioneered a multi-color staining technique geared towards discerning T_pex_ cells, marked by their TCF-1^+^PD-1^+^ phenotype, within human CRC samples. Our findings underscored that the infiltration level of T_pex_ cells was positively correlated with improved patient survival rates. Furthermore, our rigorous data mining endeavors showed that within the TME of human CRC, there was a conspicuous elevation of the granule-associated gene *GZMK* in the TCF-1^+^PD-1^+^T_pex_ cell subset, as opposed to *GZMB*. Experiments using the murine MC38 tumor model further supported these insights, displaying heightened co-expression of *Ccl5*, *Cxcr3*, *Cxcr4*, and *Eomes* in the TCF-1^+^PD-1^+^Gzmk^+^T_pex_ cell subset, alongside a decline in the expression of *Tbx21* and *Cxcr6*. Notably, the pivotal role of GZMK expressed by T_pex_ cells has been highlighted in promoting the senescence-associated secretory phenotype (SASP) in fibroblasts. This, in turn, potentially augments the formation of type-1 tumor-associated neutrophils and macrophages (42).

The trajectory of our research led us to delve into ST sequencing data from CRC patients, aiming to decode the intricate interactions of T_pex_ cells with other cellular entities within the TME (20, 21, 24). These endeavors illuminated the robust and positive correlation between T_pex_ cells and TAM1, reinforcing the likelihood of their synergistic interaction and their pivotal roles within the TME.

CD8^+^T cells are pivotal in mediating the destruction of tumor cells, primarily through the granzyme/perforin and Fas/FasL pathways. Intriguingly, our observations revealed that the transcription factor *Eomes* wasn’t dominantly expressed in T_pex_ cells. In contrast, the transcription factor *Tbx21* was markedly more pronounced in T_eff_ and T_ex_ cells. Prior studies have underscored that both transcription factors EOMES and T-bet are instrumental in orchestrating the T-BOX program (37). As delineated in **Figure 1D**, our findings suggested that immunotherapy could potentially drive the differentiation of T_pex_ cells into T_eff_ and T_ex_ cells, thereby pivoting the regulatory dynamics of target genes from *Eomes* to *Tbx21*. Notably, the ratio of infiltrating T_pex_ cells within the TME has emerged as a crucial biomarker for gauging the effectiveness of anti-PD-1 therapy in a myriad of cancer patients (15). Preliminary data highlighted that, in the adoptive T-cell transfer therapy model, tumor growth was notably curtailed in the T_pex_-cell infusion group compared to the T_ex_-cell infusion cohort. Furthermore, when combined with anti-PD-1 therapy, T_pex_-cell infusion consistently produced T_ex_ cells. In contrast, the combination of T_ex_-cell infusion and anti-PD-1 therapy did not yield the same result (22).

While our findings indicate that immunotherapy resistance is correlated with little infiltrated T_pex_ cells, immunotherapy resistance in a specific group of patients with colorectal cancer liver metastases (CRLM) may be attributed to increased T_ex_ and immunosuppressive cells, including Tregs and TAM2s (43). Concurrently targeting these immunosuppressive cells while administering ICIs therapy may enhance immunotherapeutic outcomes for patients with CRLM.

The dynamic relationship between tumor cells and immune cell infiltration is pivotal in both cancer progression and tumor immunotherapy. Quiescent tumor cells uniquely cluster together, which may pose an obstacle to immune cell access within the TME. On the flip side, highly proliferative tumor cells are closely tied to an increase in immune cell infiltration. This relationship is likely driven by their escalated proliferation and metabolic activity, which may serve as a beacon to draw in and activate immune cells (27). Our mIHC staining, as illustrated in **Figure 4B**, underscored the importance of this interaction. Specifically, we discerned a marked difference in the correlation between the proportion of TCF1^+^CK cells (reflecting quiescent tumor states) and various CD8^+^T cell components, as compared to the proportion of KI67^+^CK cells. This divergence suggested that the presence of TCF1^+^CK cells might deter CD8^+^T cell infiltration, potentially facilitating immune evasion and influencing tumor immunotherapy outcomes. In essence, our research unraveled the intricate relationship between varying tumor cell states and immune cell infiltration, offering invaluable insights for targeting therapeutic interventions. Such insights aim to amplify immune responses against tumors, ultimately enhancing treatment efficacy.

## Data availability statement

The datasets presented in this study can be found in online repositories. The names of the repository/repositories and accession number(s) can be found in the article/**Supplementary Material**.

## Ethics statement

The studies involving humans were approved by the Clinical Research Ethics Committee at Outdo Biotech (Shanghai, China, SHYJS-CP-1707004). The studies were conducted in accordance with the local legislation and institutional requirements. The participants provided their written informed consent to participate in this study.

## Author contributions

HH: Data curation, Investigation, Methodology, Writing – original draft, Writing – review & editing. JG: Data curation, Methodology, Writing – original draft. ZF: Data curation, Writing – original draft. SW: Data curation, Writing – original draft. HJ: Data curation, Writing – original draft. YYL: Data curation, Writing – original draft. JC: Data curation, Writing – original draft. WX: Data curation, Writing – original draft. BX: Data curation, Writing – original draft. YTL: Data curation, Writing – original draft. LC: Conceptualization, Project administration, Writing – original draft. XZ: Conceptualization, Project administration, Writing – original draft. JJ: Conceptualization, Funding acquisition, Project administration, Writing – original draft.

## Supplementary material

Supplementary Figure 1 mIHC based on the TMA of CRC

Supplementary Figure 2 The proportion of proliferating CD8^+^T cell infiltration is associated with the prognosis of CRC patients **(A)** mIHC and single-color images were obtained from CRC TMA, staining KI67^+^CD8^+^T cells. DAPI: light blue, CK: dark green, CD8: yellow, KI67: purple. **(B)** Kaplan-Meier survival analysis of the percentage of KI67^+^CD8^+^T cells among CD8^+^T cells in CRC patients. **(C)** mIHC and single-color images were obtained from CRC TMA, staining KI67^+^ T_pex_ cells. DAPI: light blue, CK: dark green, CD8: yellow, KI67: purple, PD-1: sky blue. **(D)** Kaplan-Meier survival analysis of the percentage of KI67^+^TCF1^+^PD-1^+^CD8^+^T cells among CD8^+^T cells in CRC patients. **(E)** Kaplan-Meier survival analysis of the percentage of KI67^+^TCF1^+^PD-1^+^CD8^+^T cells among TCF1^+^PD-1^+^CD8^+^T cells in CRC patients. **(F)** Kaplan-Meier survival analysis of the percentage of KI67^+^TCF1^+^PD-1^+^CD8^+^T cells among KI67^+^CD8^+^T cells in CRC patients. **(B, D–F)** The high expression group and low expression group were defined by the median values of percentages, and the *P* values for the differences between the high- and low-expression groups were calculated by using the Kaplan-Meier test, whereas *HR* and 95%*CI* using univariate Cox’s regression.

Supplementary Figure 3 The proportions of CD8^+^T cell subsets between CRC tissues and normal colorectal tissues **(A)** Comparison of the proportions of CD8^+^T cell subsets between CRC tissues and normal colorectal tissues. **(B)** Comparison of the proportions of TCF1^+^PD-1^+^CD8^+^T cell subsets between CRC tissues and normal colorectal tissues. **(C)** Comparison of the proportions of KI67^+^TCF1^+^PD-1^+^CD8^+^T cell subsets between CRC tissues and normal colorectal tissues. **(A–C)**Statistical analyses were performed using an unpaired, two-tailed Kolmogorov-Smirnov test. ^*^ *P* < 0.05, ^**^ *P* < 0.01, ^***^ *P* < 0.001, ^****^ *P* < 0.0001.

Supplementary Figure 4 The proportion of proliferating epithelial cell infiltration is associated with the prognosis of CRC patients **(A)** mIHC and single-color images were obtained from CRC TMA, staining TCF1^+^CK^+^ and KI67^+^CK^+^ cells. DAPI: light blue; CK: dark green; KI67: purple; TCF1: red. **(B)** Kaplan-Meier survival analysis of the percentage of CK^+^ cells in CRC patients. **(C)** Kaplan-Meier survival analysis of the percentage of TCF1^+^CK^+^ cells in CRC patients. **(D)** Kaplan-Meier survival analysis of the percentage of KI67^+^CK^+^ cells in CRC patients. **(B–D)** The high-expression group and low-expression group were defined by the median values of percentages, and the *P* values for the differences between the high- and low-expression groups were calculated by using the Kaplan–Meier test, whereas *HR* and 95%*CI* using univariate Cox’s regression.

Supplementary Figure 5 Predicted T_pex_ cells and TAM1 distribution by deconvoluting the spatially-indexed dataset

Supplementary Figure 6 T_pex_ cells can predict the therapeutic potential of melanoma cancer immunotherapy Statistical analyses were performed using unpaired, two-tailed Student’s *t*-test.
