# Supplementary figures and images for: Precursor exhausted CD8+T cells in colorectal cancer tissues associated with patient’s survival and immunotherapy responsiveness
Source: Front Immunol. 2024 Mar 6;15:1362140. doi: 10.3389/fimmu.2024.1362140 (PMC10950923; doi:10.3389/fimmu.2024.1362140)

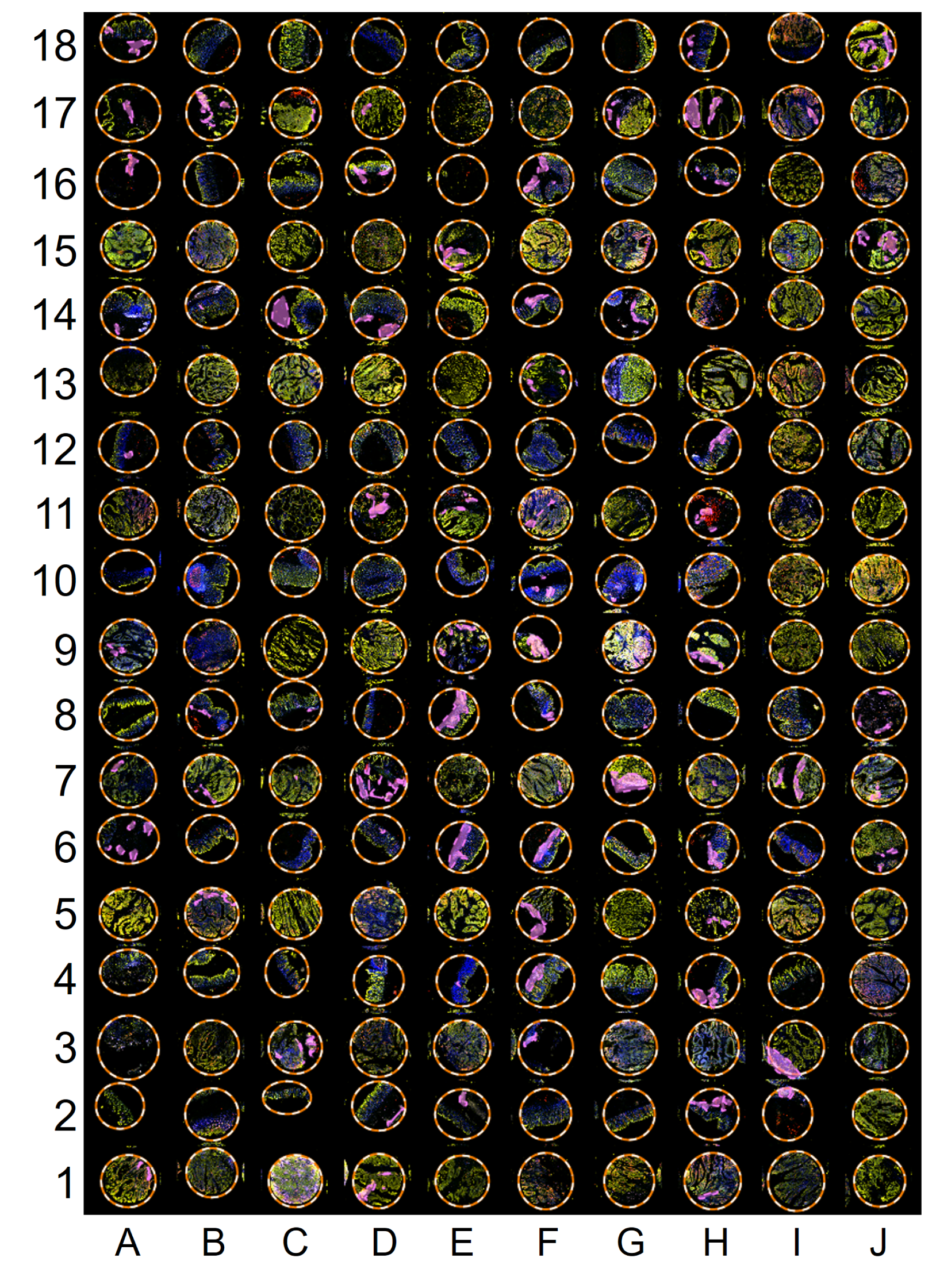

Supplement: Supplementary Figure 1 — mIHC based on the TMA of CRC [file Image_1.tif]

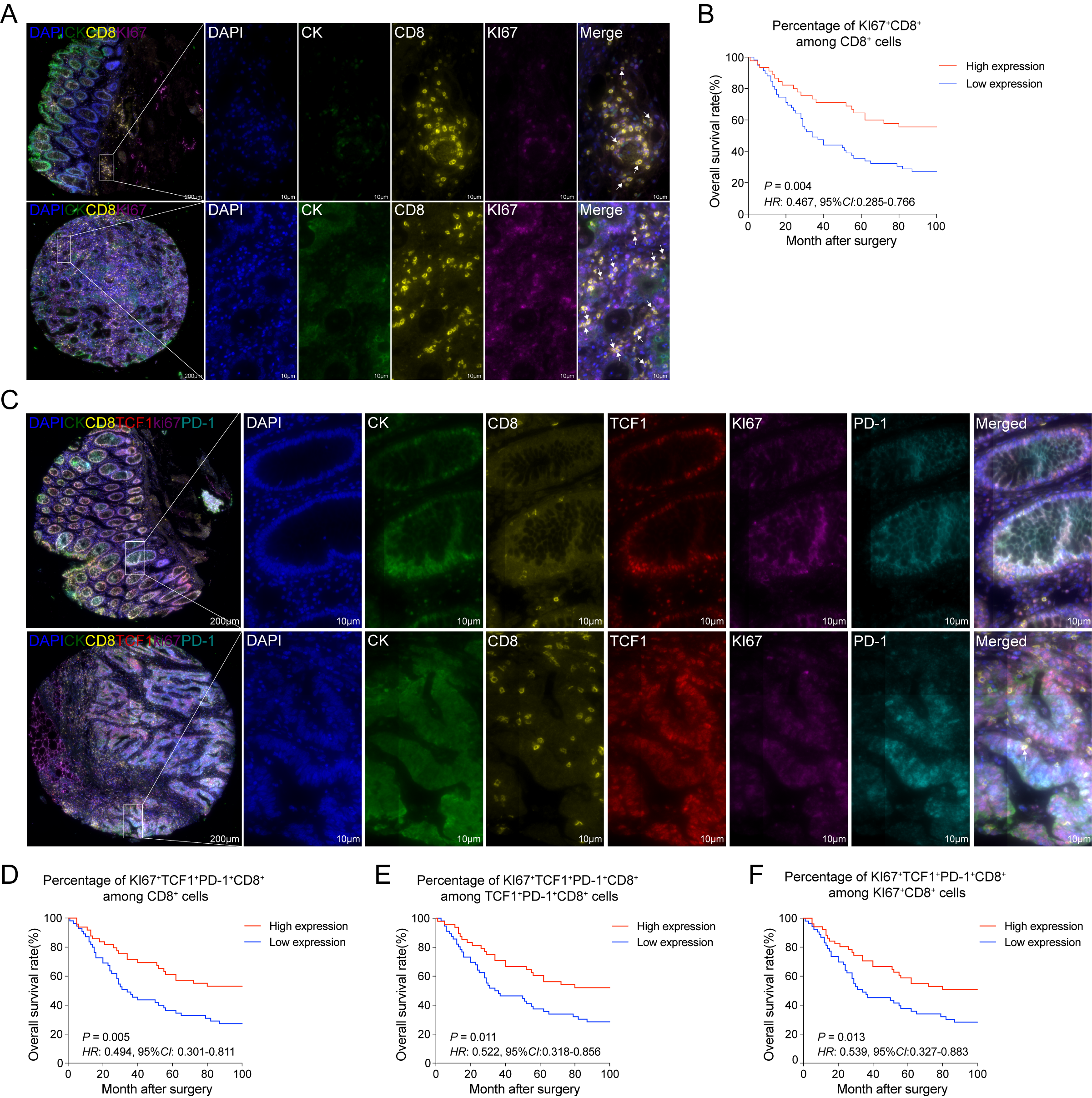

Supplement: Supplementary Figure 2 — The proportion of proliferating CD8+T cell infiltration is associated with the prognosis of CRC patients (A) mIHC and single-color images were obtained from CRC TMA, staining KI67+CD8+T cells. DAPI: light blue, CK: dark green, CD8: yellow, KI67: purple. (B) Kaplan-Meier survival analysis of the percentage of KI67+CD8+T cells among CD8+T cells in CRC patients. (C) mIHC and single-color images were obtained from CRC TMA, staining KI67+ Tpex cells. DAPI: light blue, CK: dark green, CD8: yellow, KI67: purple, PD-1: sky blue. (D) Kaplan-Meier survival analysis of the percentage of KI67+TCF1+PD-1+CD8+T cells among CD8+T cells in CRC patients. (E) Kaplan-Meier survival analysis of the percentage of KI67+TCF1+PD-1+CD8+T cells among TCF1+PD-1+CD8+T cells in CRC patients. (F) Kaplan-Meier survival analysis of the percentage of KI67+TCF1+PD-1+CD8+T cells among KI67+CD8+T cells in CRC patients. (B, D–F) The high expression group and low expression group were defined by the median values of percentages, and the P values for the differences between the high- and low-expression groups were calculated by using the Kaplan-Meier test, whereas HR and 95%CI using univariate Cox’s regression. [file Image_2.tif]

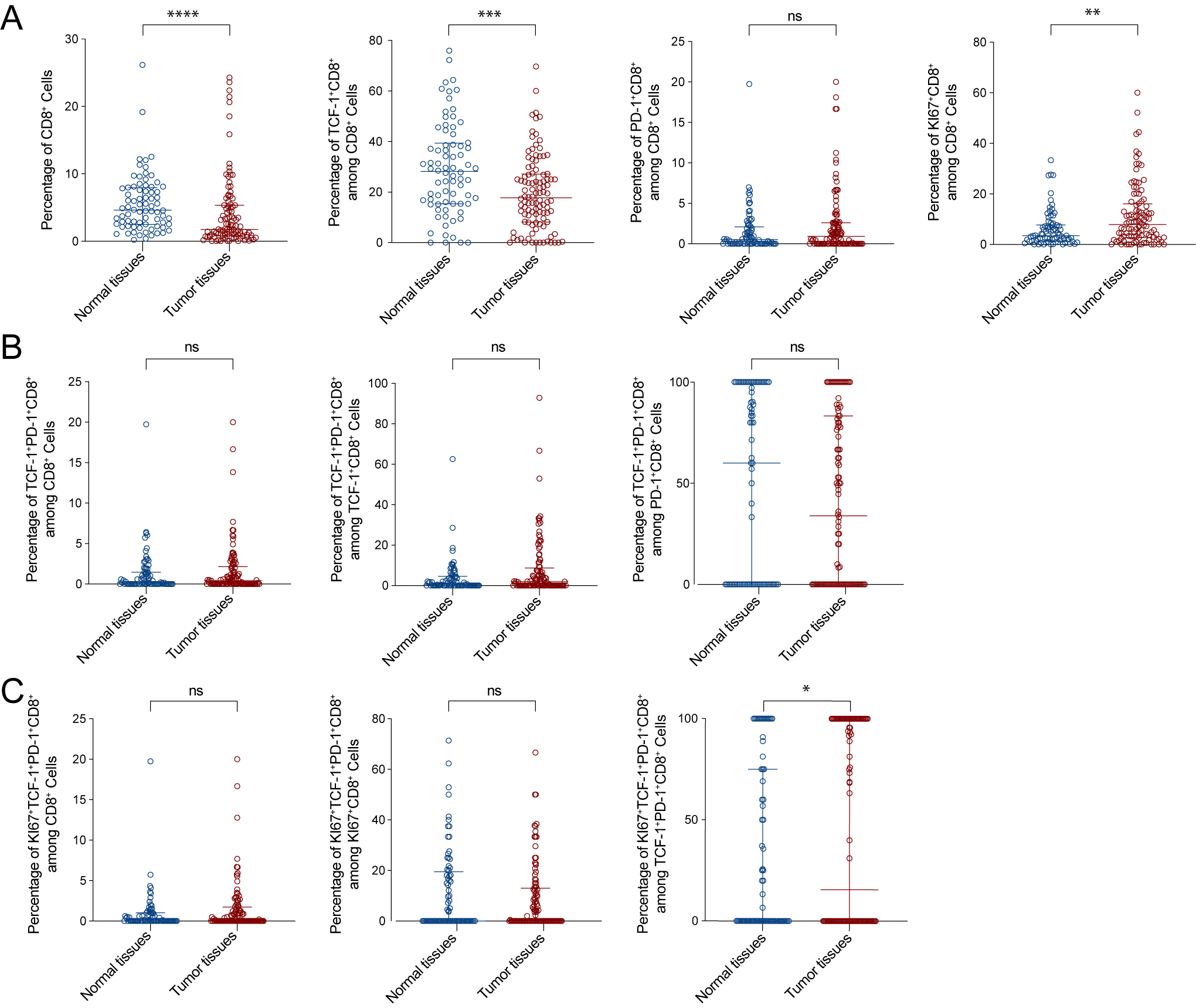

Supplement: Supplementary Figure 3 — The proportions of CD8+T cell subsets between CRC tissues and normal colorectal tissues (A) Comparison of the proportions of CD8+T cell subsets between CRC tissues and normal colorectal tissues. (B) Comparison of the proportions of TCF1+PD-1+CD8+T cell subsets between CRC tissues and normal colorectal tissues. (C) Comparison of the proportions of KI67+TCF1+PD-1+CD8+T cell subsets between CRC tissues and normal colorectal tissues. (A–C)Statistical analyses were performed using an unpaired, two-tailed Kolmogorov-Smirnov test. * P < 0.05, ** P < 0.01, *** P < 0.001, **** P < 0.0001. [file Image_3.tif]

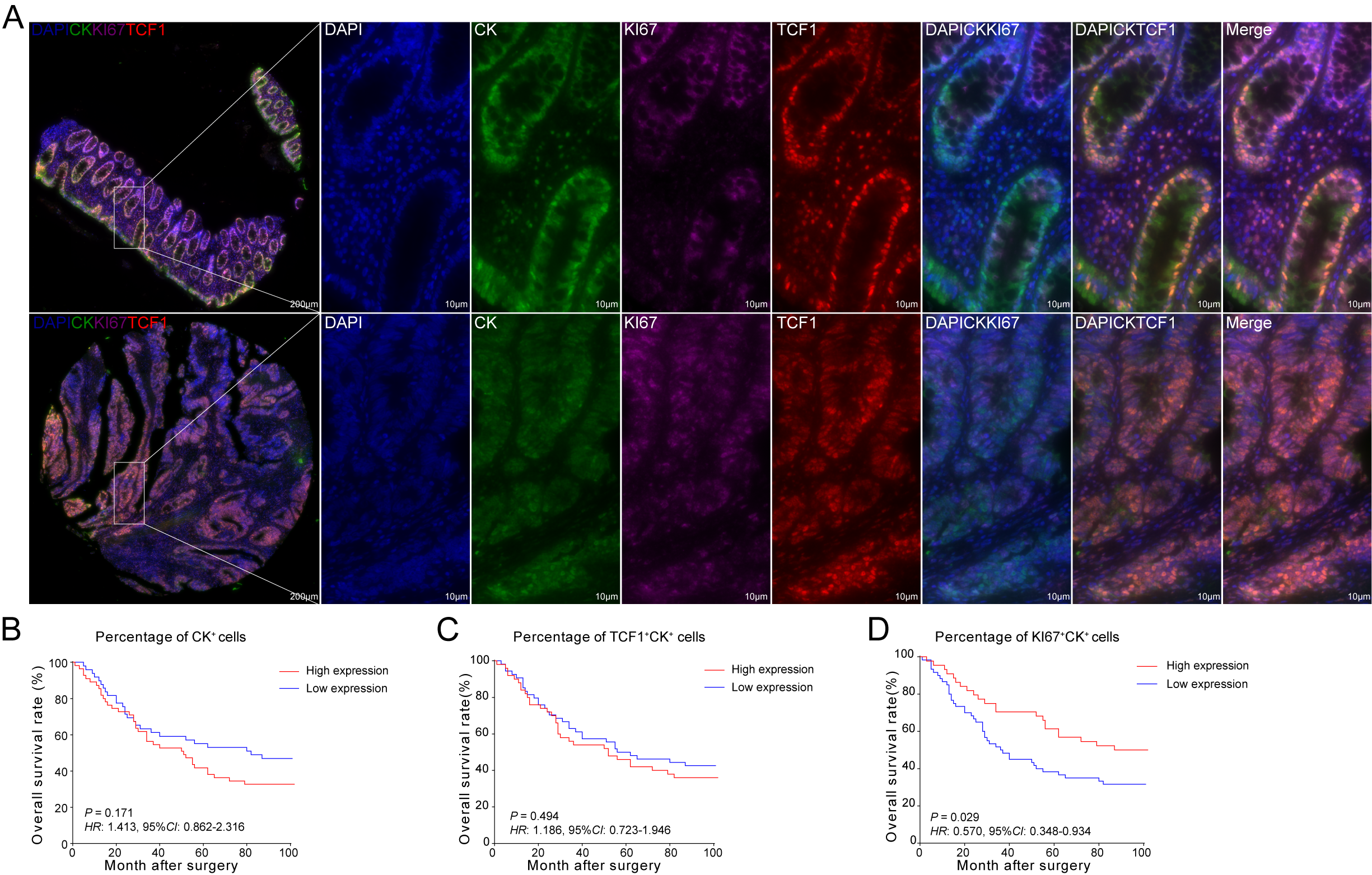

Supplement: Supplementary Figure 4 — The proportion of proliferating epithelial cell infiltration is associated with the prognosis of CRC patients (A) mIHC and single-color images were obtained from CRC TMA, staining TCF1+CK+ and KI67+CK+ cells. DAPI: light blue; CK: dark green; KI67: purple; TCF1: red. (B) Kaplan-Meier survival analysis of the percentage of CK+ cells in CRC patients. (C) Kaplan-Meier survival analysis of the percentage of TCF1+CK+ cells in CRC patients. (D) Kaplan-Meier survival analysis of the percentage of KI67+CK+ cells in CRC patients. (B–D) The high-expression group and low-expression group were defined by the median values of percentages, and the P values for the differences between the high- and low-expression groups were calculated by using the Kaplan–Meier test, whereas HR and 95%CI using univariate Cox’s regression. [file Image_4.tif]

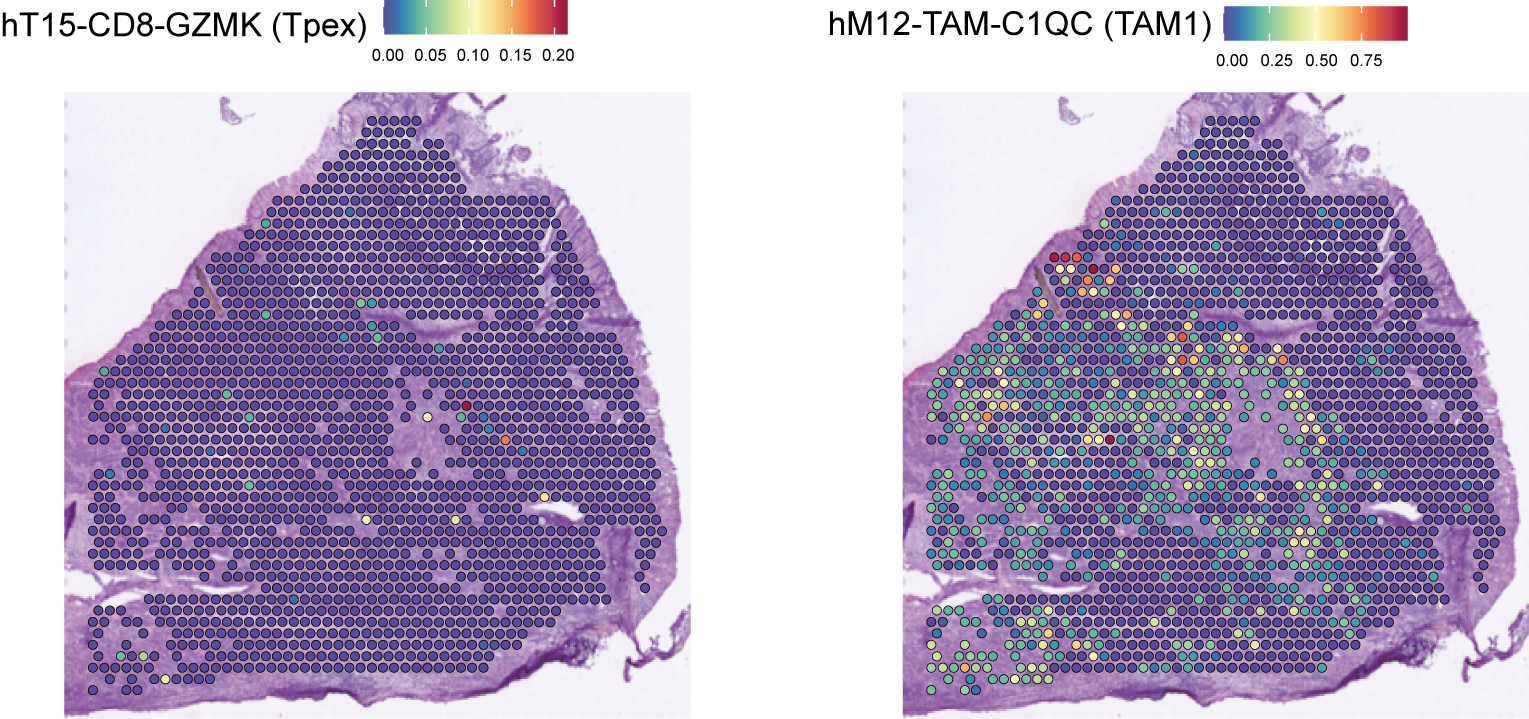

Supplement: Supplementary Figure 5 — Predicted Tpex cells and TAM1 distribution by deconvoluting the spatially-indexed dataset [file Image_5.tif]

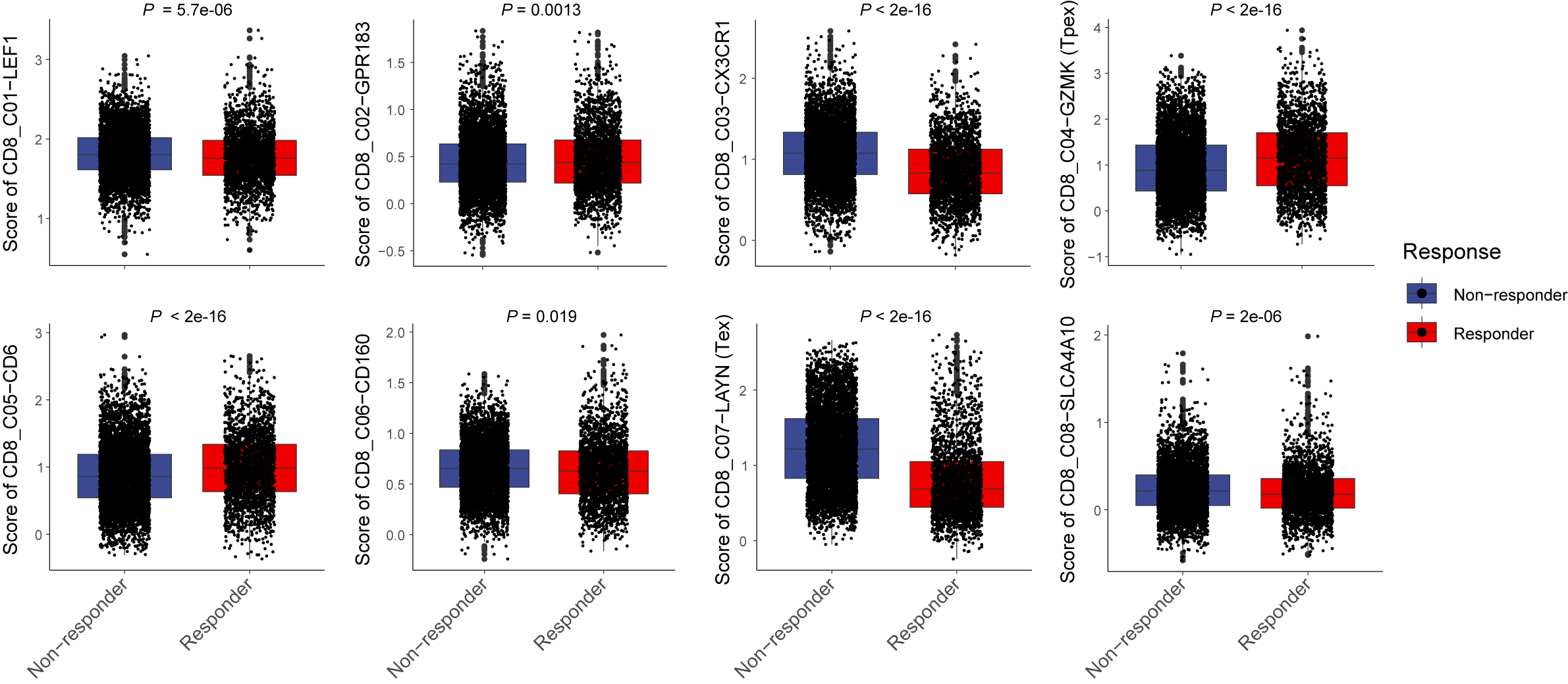

Supplement: Supplementary Figure 6 — Tpex cells can predict the therapeutic potential of melanoma cancer immunotherapy Statistical analyses were performed using unpaired, two-tailed Student’s t-test. [file Image_6.tif]
